# Expanding the Mutation Spectrum in *ABCA4*: Sixty Novel Disease Causing Variants and Their Associated Phenotype in a Large French Stargardt Cohort

**DOI:** 10.3390/ijms19082196

**Published:** 2018-07-27

**Authors:** Marco Nassisi, Saddek Mohand-Saïd, Claire-Marie Dhaenens, Fiona Boyard, Vanessa Démontant, Camille Andrieu, Aline Antonio, Christel Condroyer, Marine Foussard, Cécile Méjécase, Chiara Maria Eandi, José-Alain Sahel, Christina Zeitz, Isabelle Audo

**Affiliations:** 1 Sorbonne Université, INSERM, CNRS, Institut de la Vision, F-75012 Paris, France; m.nassisi@gmail.com (M.N.); saddekms@gmail.com (S.M.-S.); fiona.boyard@gmail.com (F.B.); vanessa.demontant@inserm.fr (V.D.); aline.antonio@inserm.fr (A.A.); christel.condroyer@inserm.fr (C.C.); marine.foussard@inserm.fr (M.F.); cecile.mejecase@inserm.fr (C.M.); j.sahel@gmail.com (J.-A.S.); 2 Department of Surgical Sciences, Eye Clinic, University of Turin, 10126 Turin, Italy; chiara.eandi@unito.it; 3 Centre Hospitalier National d’Ophtalmologie des Quinze-Vingts, DHU Sight Restore, INSERM-DHOS CIC 1423, F-75012 Paris, France; candrieu@15-20.fr; 4 Univ. Lille, Inserm UMR-S 1172, CHU Lille, Biochemistry and Molecular Biology Department—UF Génopathies, F-59000 Lille, France; claire-marie.dhaenens@chru-lille.fr; 5 Fondation Ophtalmologique Adolphe de Rothschild, F-75019 Paris, France; 6 Académie des Sciences-Institut de France, F-75006 Paris, France; 7 Department of Ophthalmology, The University of Pittsburgh School of Medicine, Pittsburg, PA 15213, USA; 8 Institute of Ophthalmology, University College of London, London EC1V 9EL, UK

**Keywords:** *ABCA4*, Stargardt disease, phenotype-genotype correlation

## Abstract

Here we report novel mutations in *ABCA4* with the underlying phenotype in a large French cohort with autosomal recessive Stargardt disease. The DNA samples of 397 index subjects were analyzed in exons and flanking intronic regions of *ABCA4* (NM_000350.2) by microarray analysis and direct Sanger sequencing. At the end of the screening, at least two likely pathogenic mutations were found in 302 patients (76.1%) while 95 remained unsolved: 40 (10.1%) with no variants identified, 52 (13.1%) with one heterozygous mutation, and 3 (0.7%) with at least one variant of uncertain significance (VUS). Sixty-three novel variants were identified in the cohort. Three of them were variants of uncertain significance. The other 60 mutations were classified as likely pathogenic or pathogenic, and were identified in 61 patients (15.4%). The majority of those were missense (55%) followed by frameshift and nonsense (30%), intronic (11.7%) variants, and in-frame deletions (3.3%). Only patients with variants never reported in literature were further analyzed herein. Recruited subjects underwent complete ophthalmic examination including best corrected visual acuity, kinetic and static perimetry, color vision test, full-field and multifocal electroretinography, color fundus photography, short-wavelength and near-infrared fundus autofluorescence imaging, and spectral domain optical coherence tomography. Clinical evaluation of each subject confirms the tendency that truncating mutations lead to a more severe phenotype with electroretinogram (ERG) impairment (*p* = 0.002) and an earlier age of onset (*p* = 0.037). Our study further expands the mutation spectrum in the exonic and flanking regions of *ABCA4* underlying Stargardt disease.

## 1. Introduction

Stargardt disease-1 (STGD1, Mendelian Inheritance in Man [MIM] 248,200) is a progressive autosomal recessive macular degeneration linked to pathogenic mutations in *ABCA4* (MIM*601,691).

*ABCA4* encodes the transmembrane protein ABCA4 (ATP-binding cassette (ABC), subfamily A, member 4), located in the outer segment disk membranes of cone and rod photoreceptor cells [1,2,3]. Its loss can lead to the development of several retinal disorders including STGD1, cone and cone-rod dystrophy, and retinitis pigmentosa [4,5,6,7,8,9,10,11].

Given the large number of variants reported in *ABCA4* [12], most of them being polymorphisms, the identification of true disease-causing mutations is often challenging [5,13,14].

With the advent of new analytical approaches, such as next generation sequencing (NGS), the detection rates of *ABCA4* mutations have greatly increased since its discovery in 1997. Nevertheless, homozygous or compound heterozygous mutations are regularly detected in no more than 70–75% of STGD1 patients, while a significant number of patients carry only a single *ABCA4* mutation or none at all [15,16,17,18,19]. On the other hand, several deep intronic variants in non-coding regions were reported to segregate with STGD1 affecting correct splicing mechanisms [19,20,21,22,23].

Interestingly, all genotyping studies on *ABCA4* report a broad mutation spectrum and a high allelic heterogeneity [6,12,24,25,26]. This phenomenon may be partially explained by the ethnic variability of the studied populations [12]. Therefore the need to screen large populations and to identify novel variants is still relevant [27,28] and can help not only to explore the genetic architecture of *ABCA4* pathology, but also the genotype/phenotype correlation. Much effort today is directed towards a more specific correlation tailored on single variants [24,28,29,30,31], even though their low frequency makes it challenging. Reporting *ABCA4* novel variants with a clear and definite correlation with STGD1 would also help the proper selection of patients for therapeutic clinical trials reaching a higher degree of confidence with molecular diagnosis [32,33].

To further explore the genetic characteristics of *ABCA4* and broaden the spectrum of its pathogenic variants, we analyzed a large French cohort of 397 STGD1 patients using a combination of microarray analysis and Sanger sequencing. The purpose of this study was to report all the novel variants identified evaluating their pathogenicity with in silico analysis. The clinical features were also analyzed and correlated with the genetic results.

## 2. Results

**Figure 1:**
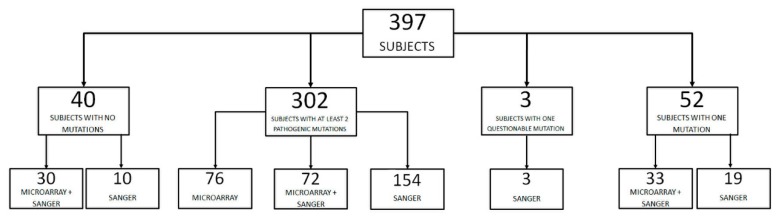
Genotypic analysis of a cohort of patients with clinical diagnosis of Stargardt disease using microarray analysis and/or direct Sanger sequencing of exonic and exon-flanking regions of *ABCA4*.

Sequencing of *ABCA4* in our cohort of 397 STGD1 patients identified a large number of allelic variants. Microarray technology (ABCR600, ASPER Biotech, Inc., Tartu, Estonia) was used to screen 211 subjects for already known mutations, demonstrating at least two pathogenic variants in 76 of them. All the unsolved and the remaining cases were addressed to direct Sanger sequencing. At the end of the screening at least two likely pathogenic mutations were found in 302 patients (76.1%), while 95 remained unsolved: 40 (10.1%) with no variants identified, 52 (13.1%) with one heterozygous mutation, and 3 (0.7%) with at least one variant of uncertain significance (VUS) (Figure 1).

### 2.1. Novel Variants

Sixty-three novel *ABCA4* variants were identified in 63 index patients (30 males); 60 variants (in 61 subjects) were likely pathogenic or pathogenic. The other 3 variants (in 3 subjects) were VUS (Table 1, uncertain). CIC03678 was a carrier of two novel mutations, one likely pathogenic and one VUS.

The 60 likely pathogenic or pathogenic variants comprised 33 missense and 6 nonsense variants, 11 deletions, 1 one-base pair (bp) duplication, 1 four-bp duplication, 1 insertion, and 7 variants presumably affecting splicing (5 single nucleotide substitutions, 1 single-bp deletion, and 1 eight-bp deletion) (Table 1), which co-segregated with the phenotype in tested available family members.

Co-segregation analysis allowed the identification of complex alleles with in cis mutations in 18 patients. Nine additional patients had more than two variations, but it was impossible to establish phase and determine each allele due to the lack of DNA from additional family members.

All genotypic information about patients and co-segregation analysis are reported in Table 2.

Four missense mutations (i.e., p.(Cys519Tyr), p.(Glu1271Gln), p.(Gly1592Asp), and p.(Asp2095Gly)) were analyzed also with the splicing predictor tools due to their proximity with a putative splice site. None of them was predicted to affect splicing significantly but they were predicted as disease-causing by the missense prediction in silico algorithms.

Among all novel mutations, p.(Tyr340Cys), p.(Val434Gly), and p.(Gly2146Valfs*36) were found at a homozygous state (Table 2).

CIC08932 inherited the complex allele p.(Asn96Lys; Gly1961Glu) from the unaffected mother, but the novel variant p.(Gly1592Asp) was not found in the unaffected father (Table 2). This variant could be de novo in the index patient or CIC08933 may not be the biological father. Unfortunately, it was impossible to get this information from the subject, and our informed consent did not cover single nucleotide polymorphisms analysis for paternity testing.

Three novel variants found in three index patients (p.(Asn956Lys), p.(Arg1137Gly), and c.5899−3T>C) were considered VUS. Conservation and pathogenic prediction data for each variant are reported in Supplementary Tables S1 and S2.

**Figure 2:**
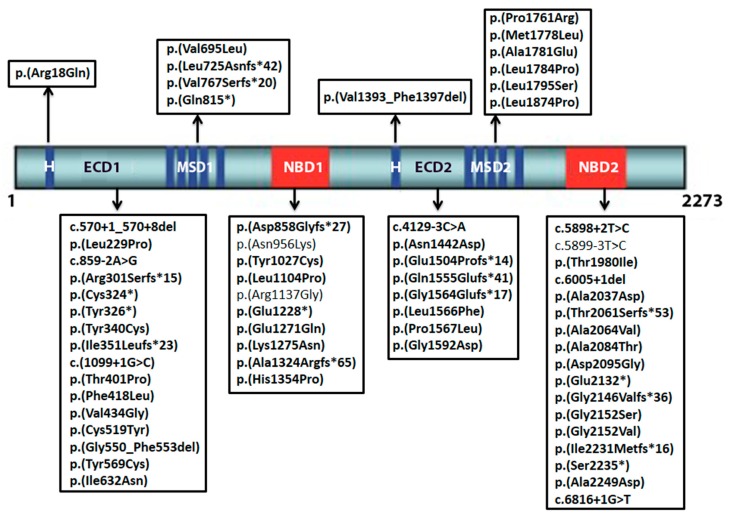
Location of novel *ABCA4* variants identified in the study. Nucleotide positions and translation correspond to CCDS747.1 and NP_000341.2, respectively. Only the three mutations of uncertain significance are not in bold letters; ECD1: first extracellular domain; NBD1: first nucleotide binding domain; MSD1: first membrane spanning domain; H: hydrophobic domain; ECD2: second extracellular domain; NBD2: second nucleotide binding domain; MSD2: second membrane spanning domain.

All novel variants found in the study and their respective position on *ABCA4* are represented in Figure 2.

### 2.2. Genotype-Phenotype Correlation

Genotype-phenotype correlation analysis was performed on the 60 patients carrying at least one likely pathogenic variant on each allele. The three subjects carrying a VUS were excluded from the analysis. All clinical features are presented in the Supplementary Table S3.

Patients were divided in two genotype groups: (1) patients with at least one null mutation (NM), i.e., frame-shift or nonsense mutations, splicing variants affecting the first two nucleotides adjacent to an exonic sequence and/or other variants proven to cause a premature stop or incomplete formation of the whole protein in previous studies (e.g., c.5461−10T>C [51]); (2) patients with two or more missense variants (MM) [52].

The two genotypic groups were not statistically different for age (mean of 34.3 ± 17.8 years for NM group and 33.9 ± 18.3 years for MM group; *p* = 0.94) nor duration of disease (mean of 16.9 ± 14.6 years for NM group and 10.8 ± 10.9 years for MM group; *p* = 0.095).

Mean age of onset was 18.5 ± 12.59 years and 23.1 ± 14.8 years in NM and MM groups, respectively (*p* = 0.037).

Right eye (RE) and left eye (LE) did not differ significantly for best corrected visual acuity (BCVA) (*p* = 0.81), central retinal thickness (CRT) (*p* = 0.09), and macular volume (MV) (*p* = 0.137), therefore we chose to use data from the right eye for comparison between genotypic groups.

**Figure 3:**
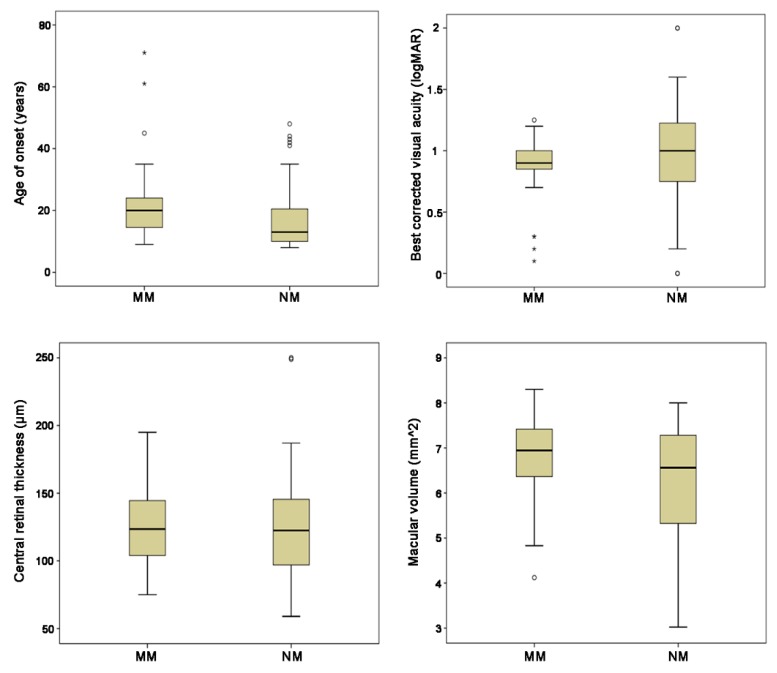
Box-plots showing the comparison of age of onset (AO), best corrected visual acuity (BCVA), central retinal thickness (CRT), and macular volume (MV) for the two genotype groups (patients with at least one null mutation (NM) and patients with two or more missense variants (MM)). The dark line in the middle of the boxes is the median; the bottom of the box indicates the 25th percentile while the top of the box represents the 75th percentile. The T-bars that extend from the boxes represent the minimum and maximum values when they are within 1.5 times the height of the box. The circles are outliers (i.e., values that do not fall in the T-bars). The stars are extreme outliers (i.e., values more than three times the height of the boxes). Only AO shows a statistically significant difference between NM and MM.

Null mutation and MM groups did not differ significantly for optical coherence tomography (OCT) parameters: mean CRT was 126.7 ± 45.3 µm and 122 ± 26.1 µm, respectively (*p* = 0.89), while mean MV was 6.2 ± 1.3 mm^2^ and 6.8 ± 1.1 mm^2^, respectively (*p* = 0.13 for MV). Mean BCVA was 0.97 ± 0.48 logMAR in the NM group and 0.85 ± 0.29 logMAR in the MM group (*p* = 0.26) (Figure 3).

The phenotype was classified following all clinical criteria summarized in Table 3.

**Figure 4:**
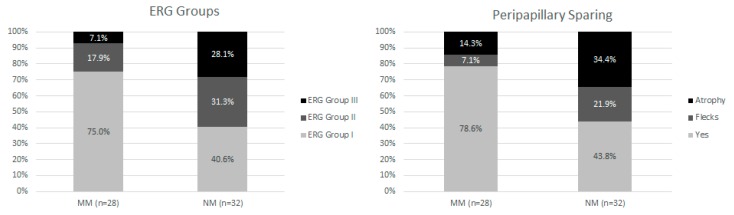
Repartition between the two genotypic subtypes for electroretinography (ERG) groups (**on the left**) and peripapillary sparing (**on the right**). For ERG classification, group I has abnormal multifocal (mf-) ERG with normal full-field (ff-) ERG; in group II there were mf-ERG abnormalities with cone dysfunction (assessed with light-adapted 30 Hz flicker and light-adapted 3.0); Group III has additional rod dysfunction (assessed using dark-adapted 0.01 and dark-adapted 10.0). Peripapillary area was considered speared (Yes in the graph) if no alterations were found in the fundus autofluorescence within 0.6 mm from the optic disc edge. This area was not considered speared when flecks or ellipsoid zone (EZ) and/or retinal pigment epithelium (RPE) atrophy were present (see Table 3 for classifications). Photoreceptors dysfunction (ERG groups II and III) and peripapillary area involved with the presence of flecks or atrophy are more prevalent in the group of patients with at least one null mutation.

Fundus staging, short-wavelength fundus autofluorescence (SW-FAF), and near infrared fundus autofluorescence (IR-FAF) groups, foveal sparing, the presence of retinal pigment epithelium (RPE) atrophy and the integrity of the ellipsoid zone (EZ) band were not statistically different between the NM and MM groups (*p* = 0.315, *p* = 0.855, *p* = 0.441, *p* = 0.327, *p* = 0.089, respectively). However, electroretinogram (ERG) group distribution and the presence of autofluorescence peripapillary sparing significantly differed between the two groups (*p* = 0.002 and *p* = 0.003 respectively) (Figure 4).

## 3. Discussion

This work is a longitudinal study starting in 2007 which used a combination of microarray analysis and direct Sanger sequencing to identify *ABCA4* mutations in a large French cohort with a clinical diagnosis of STGD1 disease. At that time targeted NGS and whole exome sequencing (WES) was not available in our laboratory. The rate of bi-allelic variants detected in the population using this approach was 75.6%, which is in accordance with previously reported data (about 75%) [15,16,17,18,19].

The authors believe that the same results would have been obtained using only direct Sanger sequencing on the entire cohort. Initially, patients were analyzed with microarray to evaluate the prevalence of known mutations, which was at this time commonly used to reduce screening costs. However, the high number of unsolved cases led us to further investigate the cohort with direct Sanger sequencing. The sole application of microarray was enough to solve 76 cases, which were not further investigated with Sanger technique. Indeed, this may have led to an underestimation of the prevalence of novel additional mutations and/or complex alleles in this cohort [28,58]. The relatively high number of unsolved cases may be related to a combination of different factors: (1) The clinical overlap of distinct genetic entities could have led to uncertainties in patients’ selection. Therefore the screening of other genes (e.g., *ELOVL4*, *PROM1*, *PRPH2*, etc.) responsible for overlapping phenotypes may help solving such cases; (2) Large rearrangements within the *ABCA4* locus have been shown to occur in ~0.5% to ~2% of STGD1 cases in previous reports and they would not have been detected by our mutational screening [22,24]; (3) Mutations in noncoding regions of the *ABCA4* gene locus have been proposed as a common source for a second causative mutation in patients with typical Stargardt phenotype. In particular, the occurrence of deep intronic variants in subjects with a single *ABCA4* mutations may range from ~2% to ~18% in different reports [19,20,21,22,27,59]. This may have led not only to an underestimation of bi-allelic cases, but also of the number of novel variants found in the cohort, whose report was the main aim of this study. However, the number of novel variants affecting deep intronic regions should be relatively small. Schulz et al. [27] in a recent report screened for deep intronic variants 237 STGD1 patients showing a single *ABCA4* variant. Among the cohort, ten different sequence variants were found, only two of which were not previously reported [27]. The addition of the first, the screening of selected noncoding regions known to be “hot spots” for pathogenic variants (e.g., intron 30 and 36 [27]), and second, whole genome sequencing (WGS) analysis, will be the next steps in order to raise the number of solved cases in our cohort.

Among the 305 patients with bi-allelic variants, we found a total of 240 disease-causing mutations, of which 60 (25%) were never described before (Table 2).

**Figure 5:**
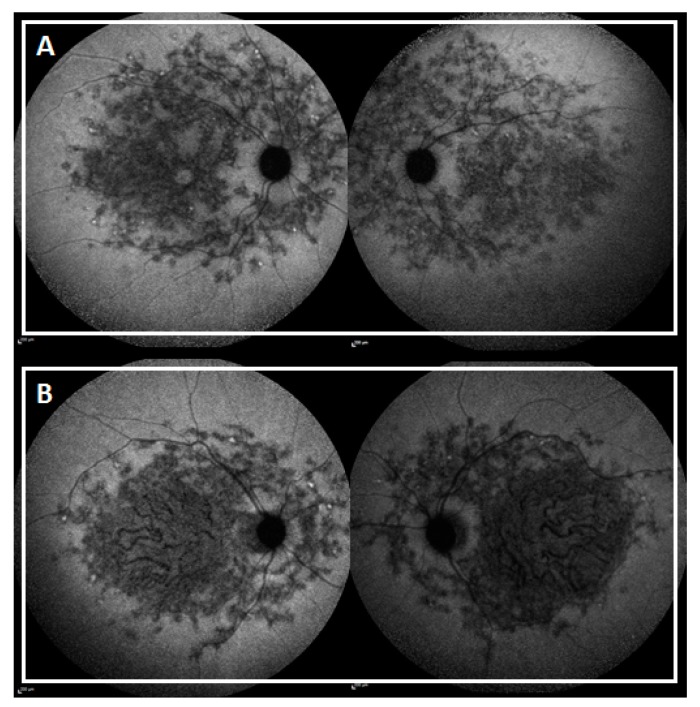
Near infrared autofluorescence (SW-AF) of subjects CIC09601 (**A**) and CIC07960 (**B**).

The high number of new variants found in this cohort may seem surprising considering that several studies had already been conducted on the western and central European populations [5,15,17,27,43]. These findings are probably related to the extensive allelic heterogeneity of *ABCA4* and the possible contribution of ethnic differences [12]. Among the 61 patients with novel likely pathogenic or pathogenic variants, three subjects had eastern European origins and nine subjects had non-European origins. The novel variant p.(Pro1761Arg) is carried by three subjects with no ties of consanguinity and with non-European origins: Armenia (CIC07960), Turkey (CIC09601), and Algeria (CIC07036). This may suggest a higher prevalence of this mutation in the south Mediterranean area. Both CIC07960 and CIC09601 harbor the variant in cis with p.(Arg2106Cys). Interestingly, the two subjects share the same pattern distribution of the lesion, but CIC07960 has an earlier age of onset and an important foveal involvement, probably due to the associated truncating mutation p.(Leu1274Serfs*8) (Figure 5).

The novel variant p.(Ile351Leufs*23) was carried by three unrelated French subjects indicating a possible higher prevalence in this population. While CIC01080 and CIC04235 share an early onset advanced disease with spread fundus atrophy, CIC07308 has a normal ff-ERG and no posterior atrophy probably due to the second mutation p.(Gly1961Glu), known for its “milder” pathologic effect [30,31].

Among the likely pathogenic novel variants three were homozygous: p.(Tyr340Cys), p.(Val434Gly), and p.(Gly2146Valfs*36).

**Figure 6:**
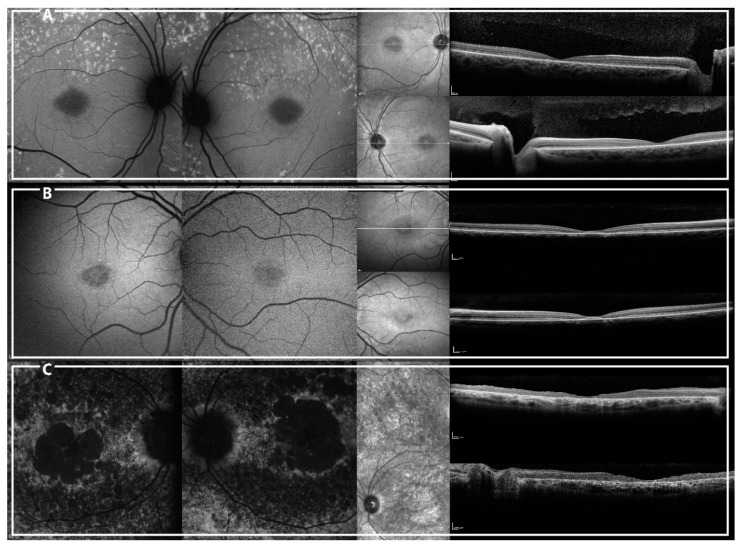
Short-wavelength autofluorescence (SW-AF) (left) and spectral domain optical coherence tomography (SD-OCT) central line (right) of patients CIC07725 (**A**), CIC09625 (**B**), and CIC06396 (**C**).

CIC07725, harboring p.(Tyr340Cys), had early-onset disease (nine years of age) with normal ff-ERG, centripetal spread of flecks and foveal EZ band involvement. CIC09625, harboring p.(Val434Gly) has an early onset disease (nine years of age) with a central lesion without flecks and with foveal sparing. Even though they share a similar age of onset and duration of the disease (one and two years, respectively), CIC09625 shows a “milder” phenotype (Figure 6). These two missense mutations may have a distinct impact on the protein function even though they are located in the same domain (first ABCA4 extracellular domain). Alternatively, genetic or environmental modifiers may explain the phenotypic variability.

As expected, CIC06396, homozygous for p.(Gly2146Valfs*36), showed a more advanced phenotype with an early onset disease (nine years of age), extensive fundus RPE atrophy and photoreceptor impairment (Figure 6).

VUS identified in the cohort were two missense variants: p.(Asn956Lys), p.(Arg1137Gly), and one non-canonical splice-site variant (c.5899−3T>C) (Table 1 and Supplementary Table S2).

CIC05824 harbors the novel intronic variant c.5899−3T>C, which is not predicted to affect splicing by the algorithms used since the nucleotide is poorly evolutionarily conserved (Supplementary Table S2). Indeed, we cannot exclude the possibility that this variant may affect splicing in vivo since it has already been proven that *ABCA4* can have non-canonical splice sites variant with important effects on protein [19,20,21,22,23,59].

The VUS p.(Asn956Lys) harbored by CIC03678 is predicted to be pathogenic only by SIFT and the AA is not conserved (Table 1, Supplementary Table S2). Asparagine and Lysine share similar characteristics of hydropathy and polarity. Unfortunately, although genetic analysis of CIC03679, unaffected son of CIC03678, revealed that he was not carrying p.(Asn956Lys), in absence of other family members available for co-segregation analysis, its significance still remains unclear.

Variant c.3409A>G, p.(Arg1137Gly) carried by CIC03545, is predicted to be pathogenic by SIFT and mutation taster although the residue was not conserved, being substituted by a Glutamine, a Threonine or a Lysine in the conservation analysis (Table 1, Supplementary Table S2). However, all these amino acids are polar while Glycine is not, hence an effect on the tridimensional structure of the protein cannot be completely excluded.

Several hypomorphic alleles have been associated with STGD1 [27,29]. Even though many of them were considered benign because of their high frequency, these alleles could be pathogenic when they are in trans with severe variants, explaining many of the unsolved cases with only one or no mutations detected on *ABCA4* [27,29]. The presence of these alleles could help in classifying accompanying *ABCA4* mutations as severe mutations and in genotype-phenotype correlations.

For example, Zernant et al. [29] recently reported that the hypormophic variant p.(Asn1868Ile) accounts for more than 50% of the missing causal alleles in monoallelic cases and about 80% of late-onset cases and distinguishes *ABCA4*-related disease from AMD [29]. In our sub-cohort of 61 patients, this variant appeared in 14 subjects. Most of these cases presented p.(Asn1868Ile) in cis with other disease causing mutations (12 subjects) and the phenotypes were typically consistent with the effect of the overall genotype with mainly early onset and severe disease as documented by Zernant et al. [29]. Only two subjects (CIC08283 and CIC04197) had p.(Asn1868Ile) alone, in trans with other pathogenic variants, resulting in a milder phenotype. Further functional analysis should be performed in order to confirm the pathogenicity of these variants.

Among the 63 patients carrying novel variants, we performed genotype-phenotype correlation on 60 of them. The three subjects carrying a VUS were excluded from the analysis. According to the genotype-phenotype model previously proposed [24], we observed an earlier age of onset and more diffuse photoreceptor dysfunction (assessed by ff-ERGs) in NM group. These findings are in accordance with the previous literature [12,52]. Regarding the peripapillary sparing, its loss was more frequent in the NM group which is also associated with a higher prevalence of non-group I ff-ERG consistent with previous publications reporting a loss of this sign in non-group I Stargardt disease [60].

Overall, in our group of 60 patients harboring novel likely pathogenic *ABCA4* changes, we observed a tendency for a worse phenotype when a truncating variant is present.

In this report we wanted to prioritize the characterization of the patients carrying novel variants. Even though it is only a subgroup of subjects the results seem strong and consistent with previous literature [12] and they probably reflect the genotype-phenotype correlation of the entire cohort. Further analysis including a full clinical assessment of patients with known variants will be necessary to confirm these findings.

However, even with a large cohort of patients, it is impossible to establish a precise phenotype-genotype correlation only on the basis of the type of the mutation. In fact, two different missense mutations involving residues at different location with distinct effects on the quaternary structure of ABCA4 can produce very different phenotypes [12,61]. Nowadays, the main efforts are aimed to find correlations between specific variants and clinical phenotype although the rarity of many alleles makes this difficult. For this reason, we tried to provide an exhaustive clinical characterization of each subject showing at least one novel mutation among the two mutant alleles of *ABCA4* (see Supplementary Table S3).

## 4. Materials and Methods

Patients with a presumed diagnosis of STGD1 disease were recruited at the Reference Center for rare diseases, Referet of the Quinze-Vingts hospital, Paris. Informed consent was obtained from each patient after explanation of the study and its potential outcome. The study protocol adhered to the tenets of the Declaration of Helsinki and was approved by a national ethics committee (CPP Ile de France V, Project number 06693, N◦EUDRACT 2006-A00347-44, 11 December 2006). All the patients and available family members were asked to donate a blood sample for genetic screening for *ABCA4* mutations. Subjects with at least one novel variant were called back to undergo a complete phenotype analysis as described below.

### 4.1. Mutation Analysis

Total genomic DNA was extracted from peripheral whole blood samples by standard salting out procedures according to the manufacturer’s recommendation (Puregen Kit; Qiagen, Courtaboeuf, France). The first consecutive 211 subjects were screened for known *ABCA4* mutations by microarray analysis on a commercially available microarray (ABCR600, ASPER Biotech, Inc., Tartu, Estonia) [48]. Among them, samples which were excluded for known variants were further investigated for variants in the coding exons and their flanking regions of *ABCA4* by PCR and direct Sanger sequencing. The DNA of the other patients included in the study was directly sequenced by Sanger sequencing. The 50 coding exons and flanking intronic regions were amplified in 48 fragments (*ABCA4* RefSeq NM_000350) using oligonucleotides reported in Supplementary Table S4, a commercially available polymerase (HotFire, Solis Biodyne, Tartu, Estonia), 1.5 mM MgCl_2_ at an annealing temperature of 58 °C for 1 min. The PCR products were enzymatically purified (ExoSAP-IT, USB Corporation, Cleveland, OH, USA, purchased from GE Healthcare, Orsay, France) sequenced and investigated as previously reported [62].

Nucleotide numbering reflects cDNA numbering with +1 corresponding to the A of the ATG translation initiation codon in the reference sequence, according to journal guidelines (www.hgvs.org/mutnomen).

All variants were classified following the American College of Medical Genetics and Genomics (ACMG) standards and guidelines and the Association of Molecular Pathology (AMP) Clinical Practice Guidelines and Reports based on previous publications (as compiled in the Human Gene Mutation Database (HGMD) [63] and in the Leiden Open (source) Variation Database (LOVD) [64]), population data, computational data and functional data. The Exome Aggregation Consortium (ExAC) database was used to check variant frequency.

Novel variants with unknown frequency or minor allele frequency (MAF) ≤0.05 were further tested using Alamut Visual software v. 2.7.1 where the in silico predictive programs PolyPhen2 (Polymorphism Phenotyping, http://genetics.bwh.harvard.edu/pph2/) [65], SIFT (Sorting Intolerant from Tolerant; http://sift.bii.a-star.edu.sg/) [66], Mutation Taster (http://www.mutationtaster.org/) [67] are implemented.

For splice site variants three different algorithms of prediction were used: MaxEntScan (http://genes.mit.edu/burgelab/maxent/Xmaxentscan_scoreseq.html) [68], NNSplice [69], and human splicing finder v 3.0 (http://www.umd.be/HSF3/) [70].

Evolutionary conservation was investigated using the 46-way Vertebrate Multiz Alignment and Conservation of the University of California Santa Cruz (UCSC) genome browser [71,72].

For missense mutations, an amino acid residue was considered highly conserved if the same residue was present in all species or was different in just one species among fishes or reptiles, moderately conserved if different in 2 to 5 species (included), and not conserved if different in more than 5 species or in at least one primate.

A novel sequence variant was considered pathogenic if it represented a nonsense variant or small insertion, deletion, or duplication inducing a frame-shift. In the case of a missense change, a novel sequence variant was considered to be likely pathogenic if it was either predicted to be deleterious by all three prediction algorithms or if it affected a highly or moderately evolutionary conserved amino acid residue and was predicted to be pathogenic by at least one algorithm mentioned above [73]. In all other cases, a variant was classified as a VUS.

The same criteria described above, but considering DNA sequence, were applied to study the conservation of nucleotide residues and pathogenicity of variants on putative or non-canonic splice sites (±10 bases from exon boundaries).

### 4.2. Phenotypic Analysis

Patients with novel *ABCA4* variants underwent full ophthalmic examination with assessment of best corrected visual acuity (BCVA) with the Early Treatment Diabetic Retinopathy Study (ETDRS) chart, kinetic and static perimetry, and color vision with the desaturated Farnsworth Panel D-15. Full-field and multifocal electroretinography (ff-ERG and mf-ERG) were performed with DTL recording electrodes and incorporated ISCEV Standards (Espion E2; for full field ERG; Diagnosys, Lowell, MA, USA; and Veris II for multifocal ERG; EDI, Redwood City, CA, USA) [74,75]. Clinical assessment was completed with color fundus photograph (FP; Topcon, Tokyo, Japan), short-wavelength fundus autofluorescence (SW-AF), near-infrared fundus autofluorescence (NIR-AF), spectral domain optical coherence tomography (SD-OCT; Heidelberg retina angiograph [HRA] II or Spectralis HRA+OCT; Heidelberg Engineering, Dossenheim, Germany) after pupil dilatation with Tropicamide 1% and phenylephrine 2.5%.

The phenotype was classified following all clinical criteria summarized in Table 3. Color fundus photographs were acquired for each index patient and stratified in 4 groups according to a previously described classification [54].

A confocal scanning laser ophthalmoscope (Heidelberg Retina Angiograph [HRA] II; Heidelberg Engineering, Dossenheim, Germany) was used to acquire SW-AF images (488 nm excitation) and NIR-AF images (787 nm excitation). Thanks to the eye-tracking function of the HRA and averaging at least 30 single frames, 2 high-quality images (30° and 50° field of view respectively) for each modality were taken for each eye. Subsequently, we divided patients into 5 groups according to a previously described classification [55]: group 1: central lesion with jagged borders, group 2: central lesion with extensive fundus changes; group 3: central lesion with smooth borders and an hyperautofluorescent ring-like halo in SW-AF and NIR-AF; group 4: central lesion with smooth borders and no hyperautofluorescent NIR-AF ring; group 5: small discrete central lesion better visualized in NIR-AF. When RPE atrophy was present the measurement of the area was performed using RegionFinder software (Heidelberg Engineering, Dossenheim, Germany; software version 2.5.8.0) on SW-AF images [76]. The 50° images were used to establish the extension of fundus abnormalities and for evaluating the peripapillary area [56].

A single horizontal high-resolution OCT B-Scan (9 mm) was obtained together with a simultaneously acquired infra-red (IR) fundus image in a transverse plane through the fovea. This image was used to evaluate the preservation of the ellipsoid zone (EZ) through the fovea [57] and the possible foveal sparing from the disease [38]. A cube of OCT scans was then obtained in high-speed mode with the automated real-time mode activated and set to 10 (creating a mean image of ten repeated identical B-scans to improve signal-to-noise ratio). The cube scan protocol contains a density of 49 B-scan lines covering an area of 20° × 20° centered on the fovea. As previously described, a trained operator corrected any segmentation errors made by the software version with manual segmentation [77,78]. When the quality of segmentation was adequate, the central retinal thickness (CRT) and the total macular volume (TMV) were recorded and compared against the data of 30 normal subjects (15 subjects younger than 30 years and 15 subjects older; Supplementary Table S5).

All patients underwent electrophysiological assessment, including ff-ERG and mf-ERG, incorporating the minimum standards of the International Society for Clinical of Electrophysiology of Vision (ISCEV) including the following stimulations: dark adapted dim flash 0.01 candela second (cd·s/m^2^); dark-adapted bright flash 10.0 cd·s/m^2^; light adapted 3.0 cd·s/m^2^ 30 Hz flicker ERG and light adapted 3.0 cd·s/m^2^ at 2 Hz. The patient data set were compared against those of 30 healthy subjects (15 younger than 30 years old and 15 older; Supplementary Table S1). The limits of ERG normality were defined for all the components of the ERG as the mean value ± 2 standard deviations. All the components of the ERG from each eye were taken into account when classifying patients into the three ERG groups defined by Lois et al. [53]: Group 1 has abnormal mf-ERG with normal ff-ERG; in Group 2 there were mf-ERG abnormalities with cone dysfunction (assessed with light-adapted 30 Hz flicker and light-adapted 3.0); Group 3 has additional rod dysfunction (assessed using dark-adapted 0.01 and dark-adapted 10.0). The overall classification was based on the more severe eye when the ERG group was different between eyes in the same patient.

### 4.3. Statistical Analysis

All data analysis was conducted using IBM SPSS Statistics software v. 21.0 (Chicago, IL, USA).

For BCVA, MV, and CRT we used the Wilcoxon signed-rank test to prove the agreement between eyes.

Differences between the two genotype groups were analyzed using the Wilcoxon-Mann-Whitney test for age of onset, age at visit, duration of the disease, BCVA, MV, and CRT. The Goodman-Kruskal Gamma test was used to analyze the difference between NM and MM groups for fundus stage, NIR-FAF, and IR-FAF groups, the presence of RPE atrophy, foveal sparing, peripapillary sparing, EZ band integrity, and ERG group distribution. For these descriptive variants, when there was discordance between the right and left eye, we considered the eye with the worse phenotype. A *p* value inferior to 0.05 was considered statistically significant.

## 5. Conclusions

Despite certain limitations in terms of detecting large CNVs (<1% of all disease-associated alleles) [79] or mutations in non-coding regions, and the lack of functional analysis, our study broadens the spectrum of *ABCA4* mutations with 60 likely pathogenic or pathogenic variants, all associated with STGD1.

## Figures and Tables

**Table 1:** Sixty-three novel variants in patients with Stargardt disease and their classification. Nucleotide positions and translation correspond to CCDS747.1 and NP_000341.2, respectively.

Exon/Intron	Variant	Protein Change	Classification
1	c.53G>A	p.(Arg18Gln)	likely pathogenic
IVS5	c.570+1_570+8del	p.?	pathogenic
6	c.686T>C	p.(Leu229Pro)	likely pathogenic
IVS7	c.859−2A>G	p.?	pathogenic
8	c.902del	p.(Arg301Serfs*15)	pathogenic
8	c.972_973delinsAT	p.(Cys324 *)	pathogenic
8	c.978C>A	p.(Tyr326 *)	pathogenic
8	c.1019A>G	p.(Tyr340Cys)	likely pathogenic
8	c.1050del	p.(Ile351Leufs*23)	pathogenic
IVS8	c.1099+1G>C	p.?	pathogenic
9	c.1201A>C	p.(Thr401Pro)	likely pathogenic
10	c.1252T>C	p.(Phe418Leu)	likely pathogenic
10	c.1301T>G	p.(Val434Gly)	likely pathogenic
12	c.1556G>A	p.(Cys519Tyr)	likely pathogenic
12	c.1648_1659del	p.(Gly550_Phe553del)	likely pathogenic
12	c.1706A>G	p.(Tyr569Cys)	likely pathogenic
13	c.1895T>A	p.(Ile632Asn)	likely pathogenic
14	c.2083G>C	p.(Val695Leu)	likely pathogenic
15	c.2169_2172dup	p.(Leu725Asnfs*42)	pathogenic
15	c.2299del	p.(Val767Serfs*20)	pathogenic
16	c.2443C>T	p.(Gln815 *)	pathogenic
16	c.2572dup	p.(Asp858Glyfs*27)	pathogenic
19	c.2868C>A	p.(Asn956Lys)	uncertain
21	c.3080A>G	p.(Tyr1027Cys)	likely pathogenic
22	c.3311T>C	p.(Leu1104Pro)	likely pathogenic
23	c.3409A>G	p.(Arg1137Gly)	uncertain
25	c.3682G>T	p.(Glu1228 *)	likely pathogenic
25	c.3811G>C	p.(Glu1271Asp)	likely pathogenic
26	c.3825G>C	p.(Lys1275Asn)	likely pathogenic
27	c.3966del	p.(Ala1324Argfs*65)	pathogenic
27	c.4061A>C	p.(His1354Pro)	likely pathogenic
IVS27	c.4129−3C>A	p.?	likely pathogenic
28	c.4178_4192del	p.(Val1393_Phe1397del)	likely pathogenic
29	c.4324A>G	p.(Asn1442Asp)	likely pathogenic
30	c.4510_4535del	p.(Glu1504Profs*42)	pathogenic
32	c.4663_4664del	p.(Gln1555Glufs*41)	pathogenic
33	c.4689del	p.(Gly1564Glufs*17)	pathogenic
33	c.4696C>T	p.(Leu1566Phe)	likely pathogenic
33	c.4700C>T	p.(Pro1567Leu)	likely pathogenic
34	c.4775G>A	p.(Gly1592Asp)	likely pathogenic
37	c.5282C>G	p.(Pro1761Arg)	likely pathogenic
38	c.5332A>T	p.(Met1778Leu)	likely pathogenic
38	c.5342C>A	p.(Ala1781Glu)	likely pathogenic
38	c.5351T>C	p.(Leu1784Pro)	likely pathogenic
38	c.5384T>C	p.(Leu1795Ser)	likely pathogenic
40	c.5621T>C	p.(Leu1874Pro)	likely pathogenic
IVS42	c.5898+2T>C	p.?	pathogenic
IVS42	c.5899−3T>C	p.?	uncertain
43	c.5939C>T	p.(Thr1980Ile)	likely pathogenic
IVS43	c.6005+1del	p.?	pathogenic
44	c.6110C>A	p.(Ala2037Asp)	likely pathogenic
45	c.6181_6184del	p.(Thr2061Serfs*53)	pathogenic
45	c.6191C>T	p.(Ala2064Val)	likely pathogenic
45	c.6250G>A	p.(Ala2084Thr)	likely pathogenic
46	c.6284A>G	p.(Asp2095Gly)	likely pathogenic
47	c.6394G>T	p.(Glu2132 *)	pathogenic
47	c.6454G>A	p.(Gly2152Ser)	likely pathogenic
47	c.6455G>T	p.(Gly2152Val)	likely pathogenic
47	c.6436_6437insT	p.(Gly2146Valfs*36)	pathogenic
48	c.6693del	p.(Ile2231Metfs*16)	pathogenic
48	c.6704C>G	p.(Ser2235 *)	pathogenic
49	c.6746C>A	p.(Ala2249Asp)	likely pathogenic
IVS49	c.6816+1G>T	p.?	pathogenic

p.?: Protein has not been analysed, an effect is expected but difficult to predict.

**Table 2:** Sixty novel likely pathogenic mutations in 61 index patients with Stargardt disease and cosegregation analysis. Nucleotide positions and translation correspond to CCDS747.1 and NP_000341.2, respectively.

			Allele 1			Allele 2			Allele 1 or 2		
Patient ID	Family ID		Exon/Intron	Nucleotide Change	Protein Change	Exon/Intron	Nucleotide Change	Protein Change	Exon/Intron	Nucleotide Change	Protein Change
CIC03734	1673	Index	1	c.53G>A	p.(Arg18Gln)	42	c.5882G>A [25]	p.(Gly1961Glu)			
CIC03735	1673	Affected brother	1	c.53G>A	p.(Arg18Gln)	42	c.5882G>A [25]	p.(Gly1961Glu)			
CIC06131	1673	Unaffected father	1	c.53G>A	p.(Arg18Gln)	WILD TYPE					
CIC06908	3783	Index	IVS5	c.570+1_570+8del	p.?	40	c.5603A>T [34]	p.(Asn1868Ile)			
			45	c.6148G>C [25]	p.(Val2050Leu)						
CIC08771	3783	Unaffected mother	WILD TYPE			40	c.5603A>T [34]	p.(Asn1868Ile)			
CIC08774	3783	Unaffected brother	IVS5	c.570+1_570+8del	-	WILD TYPE					
			45	c.6148G>C [25]	p.(Val2050Leu)						
CIC07895	4412	Index	6	c.686T>C	p.(Leu229Pro)	24	c.3602T>G [5]	p.(Leu1201Arg)			
						40	c.5621T>C	p.(Leu1874Pro)			
CIC07896	4412	Unaffected mother	6	c.686T>C	p.(Leu229Pro)	WILD TYPE					
CIC07887	4407	Index	6	c.686T>C	p.(Leu229Pro)	42	c.5882G>A [25]	p.(Gly1961Glu)			
			48	c.6529G>A [35]	p.(Asp2177Asn)						
CIC07888	4407	Unaffected mother	6	c.686T>C	p.(Leu229Pro)	WILD TYPE					
			48	c.6529G>A [35]	p.(Asp2177Asn)						
CIC09848	5658	Index	IVS7	c.859−2A>G	-	8	c.872C>T [36]	p.(Pro291Leu)			
						11	c.1531C>T [18]	p.(Arg511Cys)			
CIC09849	5658	Unaffected mother	WILD TYPE			8	c.872C>T [37]	p.(Pro291Leu)			
						11	c.1531C>T [18]	p.(Arg511Cys)			
CIC09382	5385	Index	8	c.978C>A	p.(Tyr326 *)	IVS42	c.5898+2T>C	p.?			
CIC06749	3669	Index	8	c.902del	p.(Arg301Serfs*15)	46	c.6320G>A [26]	p.(Arg2107His)			
CIC06088	3203	Index	8	c.972_973delinsAT	p.(Cys324 *)	23	c.3386G>T [38]	p.(Arg1129Leu)			
CIC08477	3203	Unaffected father	8	c.972_973delinsAT	p.(Cys324 *)	WILD TYPE					
CIC08478	3203	Unaffected mother	WILD TYPE			23	c.3386G>T [38]	p.(Arg1129Leu)			
CIC07725	4301	Index	8	c.1019A>G	p.(Tyr340Cys)	8	c.1019A>G	p.(Tyr340Cys)			
CIC07726	4301	Unaffected father	8	c.1019A>G	p.(Tyr340Cys)	WILD TYPE					
CICO7727	4301	Unaffected mother	WILD TYPE			8	c.1019A>G	p.(Tyr340Cys)			
CIC04235	2021	Index	8	c.1050del	p.(Ile351Leufs*23)	38	c.5351T>C	p.(Leu1784Pro)			
CIC04236	2021	Unaffected sister	8	c.1050del	p.(Ile351Leufs*23)	WILD TYPE					
CIC07308	4026	Index	8	c.1050del	p.(Ile351Leufs*23)	42	c.5882G>A [25]	p.(Gly1961Glu)			
CIC01080	659	Index	8	c.1050del	p.(Ile351Leufs*23)	19	c.2819C>G [39]	p.(Pro940Arg)			
						23	c.3364G>A [6]	p.(Glu1122Lys)			
CIC03521	659	Unaffected mother	WILD TYPE			19	c.2819C>G [39]	p.(Pro940Arg)			
						23	c.3364G>A [6]	p.(Glu1122Lys)			
CIC03524	659	Unaffected sister	WILD TYPE			WILD TYPE					
CIC08372	4715	Index	IVS8	c.1099+1G>C	p.?	22	c.3322C>T [26]	p.(Arg1108Cys)			
CIC05266	2672	Index	9	c.1201A>C	p.(Thr401Pro)	17	c.2588G>C [25]	p.(Gly863Ala)			
						40	c.5603A>T [34]	p.(Asn1868Ile)			
CIC05267	2672	Unaffected mother	9	c.1201A>C	p.(Thr401Pro)	WILD TYPE					
CIC05268	2672	Unaffected father	WILD TYPE			17	c.2588G>C [25]	p.(Gly863Ala)			
						40	c.5603A>T [34]	p.(Asn1868Ile)			
CIC02804	1037	Index	10	c.1252T>C	p.(Phe418Leu)	IVS43	c.6005+1del	p.?	42	c.5882G>A [25]	p.(Gly1961Glu)
CIC09625	5521	Index	10	c.1301T>G	p.(Val434Gly)	10	c.1301T>G	p.(Val434Gly)			
CIC09624	5521	Affected brother	10	c.1301T>G	p.(Val434Gly)	10	c.1301T>G	p.(Val434Gly)			
CIC09626	5521	Unaffected father	10	c.1301T>G	p.(Val434Gly)	WILD TYPE					
CIC09628	5521	Unaffected mother	WILD TYPE			10	c.1301T>G	p.(Val434Gly)			
CIC01301	784	Index	12	c.1556G>A	p.(Cys519Tyr)	22	c.3292C>T [40]	p.(Arg1098Cys)			
CIC09999	784	Affected brother	12	c.1556G>A	p.(Cys519Tyr)	22	c.3292C>T [41]	p.(Arg1098Cys)			
CIC06346	3356	Index	12	c.1648_1659del	p.(Gly550_Phe553del)	42	c.5882G>A [25]	p.(Gly1961Glu)			
CIC08884	3356	Unaffected father	12	c.1648_1659del	p.(Gly550_Phe553del)	WILD TYPE					
CIC08949	3356	Unaffected mother	WILD TYPE			42	c.5882G>A [25]	p.(Gly1961Glu)			
CIC04197	1992	Index	12	c.1706A>G	p.(Tyr569Cys)	40	c.5603A>T [34]	p.(Asn1868Ile)			
CIC07749	4314	Index	13	c.1895T>A	p.(Ile632Asn)	33	c.4700C>T	p.(Pro1567Leu)	24	c.3602T>G [5]	p.(Leu1201Arg)
CIC08724	4948	Index	14	c.2083G>C	p.(Val695Leu)	47	c.6445C>T [6]	p.(Arg2149 *)			
			44	c.6079C>T [29]	p.(Leu2027Phe)						
CIC08723	4948	Affected sister	14	c.2083G>C	p.(Val695Leu)	47	c.6445C>T [6]	p.(Arg2149 *)			
			44	c.6079C>T [29]	p.(Leu2027Phe)						
CIC08858	4948	Unaffected mother	14	c.2083G>C	p.(Val695Leu)	WILD TYPE					
			44	c.6079C>T [29]	p.(Leu2027Phe)						
CIC08859	4948	Unaffected father	WILD TYPE			47	c.6445C>T [6]	p.(Arg2149 *)			
CIC07985	4793	Index	15	c.2169_2172dup	p.(Leu725Asnfs*42)	47	c.6454G>A	p.(Gly2152Ser)	44	c.6079C>T [25]	p.(Leu2027Phe)
									40	c.5603A>T [34]	p.(Asn1868Ile)
CIC06126	3227	Index	15	c.2299del	p.(Val767Serfs*20)	17	c.2588G>C [25]	p.(Gly863Ala)	40	c.5603A>T [34]	p.(Asn1868Ile)
CIC09405	5401	Index	16	c.2443C>T	p.(Gln815 *)	42	c.5882G>A [25]	p.(Gly1961Glu)			
CIC09407	5401	Unaffected mother	WILD TYPE			42	c.5882G>A [25]	p.(Gly1961Glu)			
CIC09408	5401	Unaffected father	16	c.2443C>T	p.(Gln815 *)	WILD TYPE					
CIC00467	319	Index	16	c.2572dup	p.(Asp858Glyfs*27)	35	c.4926C>G [42]	p.(Ser1642Arg)			
						36	c.5044_5058del [25]	p.(Val1682_Val1686del)			
CIC05014	319	Unaffected sister	16	c.2572dup	p.(Asp858Glyfs*27)	WILD TYPE					
CIC05056	319	Affected brother	16	c.2572dup	p.(Asp858Glyfs*27)	35	c.4926C>G [43]	p.(Ser1642Arg)			
						36	c.5044_5058del [25]	p.(Val1682_Val1686del)			
^+^ CIC03678	1627	Index	19	c.2868C>A	p.(Asn956Lys)	43	c.5939C>T	p.(Thr1980Ile)			
CIC03679	1627	Unaffected son	WILD TYPE			43	c.5939C>T	p.(Thr1980Ile)			
CIC04259	2036	Index	21	c.3080A>G	p.(Tyr1027Cys)	38	c.5381C>A [24]	p.(Ala1794Asp)			
CIC08538	4826	Index	22	c.3311T>C	p.(Leu1104Pro)	IVS49	c.6816+1G>A [6]	-			
CIC04176	1973	Index	25	c.3682G>T	p.(Glu1228*)	22	c.3322C>T [26]	p.(Arg1108Cys)			
CIC02505	871	Index	25	c.3811G>C	p.(Glu1271Gln)	23	c.3386G>T [38]	p.(Arg1129Leu)			
CIC05899	3087	Index	25	c.3811G>C	p.(Glu1271Gln)	42	c.5882G>A [25]	p.(Gly1961Glu)			
CIC07120	3909	Index	26	c.3825G>C	p.(Lys1275Asn)	22	c.3322C>T [26]	p.(Arg1108Cys)	40	c.5603A>T [34]	p.(Asn1868Ile)
									46	c.6320G>A [26]	p.(Arg2107His)
CIC01750	1242	Index	27	c.3966del	p.(Ala1324Argfs*65)	35	c.4918C>T [26]	p.(Arg1640Trp)			
						40	c.5603A>T [34]	p.(Asn1868Ile)			
CIC02024	1242	Affected sister	27	c.3966del	p.(Ala1324Argfs*65)	35	c.4918C>T [26]	p.(Arg1640Trp)			
						40	c.5603A>T [34]	p.(Asn1868Ile)			
CIC07100	1242	Unaffected father	WILD TYPE			35	c.4918C>T [26]	p.(Arg1640Trp)			
						40	c.5603A>T [34]	p.(Asn1868Ile)			
CIC07146	1242	Unaffected mother	27	c.3966del	p.(Ala1324Argfs*65)	WILD TYPE					
CIC07744	4372	Index	27	c.4061A>C	p.(His1354Pro)	13	c.1927G>A [44]	p.(Val643Met)	8	c.872C>T [37]	p.(Pro291Leu)
									24	c.3602T>G [6]	p.(Leu1201Arg)
CIC02690	961	Index	IVS27	c.4129−3C>A	p.?	IVS38	c.5461-10T>C [45]	p. [Thr1821Valfs, Thr1821Aspfs]			
						40	c.5603A>T [34]	p.(Asn1868Ile)			
CIC02691	961	Unaffected mother	WILD TYPE			IVS38	c.5461-10T>C [45]	p. [Thr1821Valfs, Thr1821Aspfs]			
						40	c.5603A>T [34]	p.(Asn1868Ile)			
CIC08194	4588	Index	28	c.4178_4192del	p.(Val1393_Phe1397del)	40	c.5603A>T [34]	p.(Asn1868Ile)	45	c.6148G>C [25]	p.(Val2050Leu)
CIC07994	4463	Index	29	c.4324A>G	p.(Asn1442Asp)	3	c.194G>A [46]	p.(Gly65Glu)			
CIC07998	4463	Unaffected mother	WILD TYPE			3	c.194G>A [46]	p.(Gly65Glu)			
CIC06727	3652	Index	30	c.4510_4535del	p.(Glu1504Profs*42)	28	c.4139C>T [6]	p.(Pro1380Leu)			
CIC06728	3652	Unaffected father	WILD TYPE			28	c.4139C>T [6]	p.(Pro1380Leu)			
CIC06729	3652	Unaffected brother	WILD TYPE			28	c.4139C>T [6]	p.(Pro1380Leu)			
CIC06730	3652	Unaffected mother	30	c.4510_4535del	p.(Glu1504Profs*42)	WILD TYPE					
CIC08283	4647	Index	32	c.4663_4664del	p.(Gln1555Glufs*41)	40	c.5603A>T [34]	p.(Asn1868Ile)			
CIC03252	1375	Index	33	c.4689del	p.(Gly1564Glufs*17)	17	c.2588G>C [25]	p.(Gly863Ala)			
						40	c.5603A>T [34]	p.(Asn1868Ile)			
CIC03253	1375	Unaffected mother	WILD TYPE			17	c.2588G>C [25]	p.(Gly863Ala)			
						40	c.5603A>T [34]	p.(Asn1868Ile)			
CIC09219	5282	Index	33	c.4696C>T	p.(Leu1566Phe)	21	c.3056C>T [26]	p.(Thr1019Met)			
			42	c.5882G>A [25]	p.(Gly1961Glu)						
CIC10754	5282	Unaffected mother		WILD TYPE		21	c.3056C>T [26]	p.(Thr1019Met)			
CIC10755	5282	Unaffected father	33	c.4696C>T	p.(Leu1566Phe)		WILD TYPE				
			42	c.5882G>A [25]	p.(Gly1961Glu)						
CIC08932	5089	Index	34	c.4775G>A	p.(Gly1592Asp)	3	c.288C>A [47]	p.(Asn96Lys)			
						42	c.5882G>A [25]	p.(Gly1961Glu)			
^++^ CIC08933	5089	Unaffected father	WILD TYPE			WILD TYPE					
CIC08934	5089	Unaffected mother	WILD TYPE			3	c.288C>A [47]	p.(Asn96Lys)			
						42	c.5882G>A [25]	p.(Gly1961Glu)			
CIC07036	3867	Index	37	c.5282C>G	p.(Pro1761Arg)	42	c.5882G>A [25]	p.(Gly1961Glu)			
CIC07960	4447	Index	37	c.5282C>G	p.(Pro1761Arg)	26	c.3819dup [48]	p.(Leu1274Serfs*8)			
			46	c.6316C>T [25]	p.(Arg2106Cys)						
CIC07999	4447	Unaffected sister	WILD TYPE			WILD TYPE					
CIC08029	4447	Unaffected mother	37	c.5282C>G	p.(Pro1761Arg)	WILD TYPE					
			46	c.6316C>T [25]	p.(Arg2106Cys)						
CIC09601	5509	Index	37	c.5282C>G	p.(Pro1761Arg)	22	c.3279C>A [18]	p.(Asp1093Glu)			
			46	c.6316C>T [25]	p.(Arg2106Cys)						
CIC09602	5509	Unaffected daughter	37	c.5282C>G	p.(Pro1761Arg)	WILD TYPE					
			46	c.6316C>T [25]	p.(Arg2106Cys)						
CIC07831	4373	Index	38	c.5332A>T	p.(Met1778Leu)	27	c.3899G>A [44]	p.(Arg1300Gln)			
CIC08439	4759	Index	38	c.5332A>T	p.(Met1778Leu)	47	c.6394G>T	p.(Glu2132 *)			
CIC08262	4633	Index	38	c.5342C>A	p.(Ala1781Glu)	3	c.286A>G [49]	p.(Asn96Asp)			
CIC08263	4633	Unaffected mother	38	c.5342C>A	p.(Ala1781Glu)	WILD TYPE					
CIC09095	4633	Unaffected father	WILD TYPE			3	c.286A>G [49]	p.(Asn96Asp)			
CIC08359	4702	Index	38	c.5384T>C	p.(Leu1795Ser)	17	c.2588G>C [25]	p.(Gly863Ala)	40	c.5603A>T [34]	p.(Asn1868Ile)
CIC07436	4110	Index	IVS42	c.5898+2T>C	p.?	40	c.5642C>T [50]	p.(Ala1881Val)			
CIC08523	4110	Unaffected mother	WILD TYPE			40	c.5642C>T [50]	p.(Ala1881Val)			
CIC08524	4110	Unaffected father	IVS42	c.5898+2T>C	p.?	WILD TYPE					
CIC09857	5675	Index	44	c.6110C>A	p.(Ala2037Asp)	IVS38	c.5461−10T>C [45]	p. [Thr1821Valfs, Thr1821Aspfs]			
						40	c.5603A>T [34]	p.(Asn1868Ile)			
CIC09858	5675	Unaffected mother	44	c.6110C>A	p.(Ala2037Asp)	WILD TYPE					
CIC10452	5675	Unaffected father	WILD TYPE			IVS38	c.5461−10T>C [45]	p. [Thr1821Valfs, Thr1821Aspfs]			
						40	c.5603A>T [34]	p.(Asn1868Ile)			
CIC01199	719	Index	45	c.6181_6184del	p.(Thr2061Serfs*53)	42	c.5882G>A [25]	p.(Gly1961Glu)			
CIC08054	719	Affected sister	45	c.6181_6184del	p.(Thr2061Serfs*53)	42	c.5882G>A [25]	p.(Gly1961Glu)			
CIC08057	719	Unaffected father	WILD TYPE			42	c.5882G>A [25]	p.(Gly1961Glu)			
CIC8058	719	Unaffected mother	45	c.6181_6184del	p.(Thr2061Serfs*53)	WILD TYPE					
CIC03710	1657	Index	45	c.6191C>T	p.(Ala2064Val)	30	c.4537dup [44]	p.(Gln1513Profs*42)			
			40	c.5603A>T [34]	p.(Asn1868Ile)						
CIC03711	1657	Unaffected mother	45	c.6191C>T	p.(Ala2064Val)	WILD TYPE					
			40	c.5603A>T [34]	p.(Asn1868Ile)						
CIC04571	2232	Index	45	c.6250G>A	p.(Ala2084Thr)	19	c.2894A>G [25]	p.(Asn965Ser)			
			42	c.5882G>A [25]	p.(Gly1961Glu)						
CIC04572	2232	Unaffected mother	WILD TYPE			19	c.2894A>G [25]	p.(Asn965Ser)			
CIC04573	2232	Unaffected maternal aunt	WILD TYPE			19	c.2894A>G [25]	p.(Asn965Ser)			
CIC07568	2232	Unaffected father	45	c.6250G>A	p.(Ala2084Thr)	WILD TYPE					
			42	c.5882G>A [25]	p.(Gly1961Glu)						
CIC07748	4312	Index	46	c.6284A>G	p.(Asp2095Gly)	22	c.3323G>A [34]	p.(Arg1108His)			
CIC09593	4312	Unaffected mother	46	c.6284A>G	p.(Asp2095Gly)	WILD TYPE					
CIC09594	4312	Unaffected father	WILD TYPE			22	c.3323G>A [34]	p.(Arg1108His)			
CIC09374	5379	Index	47	c.6455G>T	p.(Gly2152Val)	9	c.1222C>T [43]	p.(Arg408*)			
CIC09375	5379	Unaffected mother	47	c.6455G>T	p.(Gly2152Val)	WILD TYPE					
CIC06396	3389	Index	47	c.6436_6437insT	p.(Gly2146Valfs*36)	47	c.6436_6437insT	p.( Gly2146Valfs*36)			
CIC00130	102	Index	48	c.6693del	p.(Ile2231Metfs*16)	44	c.6079C>T [25]	p.(Leu2027Phe)			
CIC00131	102	Unaffected father	48	c.6693del	p.(Ile2231Metfs*16)	WILD TYPE					
CIC00132	102	Unaffected mother	WILD TYPE			44	c.6079C>T [25]	p.(Leu2027Phe)			
CIC09114	5205	Index	48	c.6704C>G	p.(Ser2235 *)	21	c.3113C>T [24]	p.(Ala1038Val)			
CIC08581	4855	Index	49	c.6746C>A	p.(Ala2249Asp)	13	c.1819G>A [40]	p.(Gly607Arg)	42	c.5882G>A [25]	p.(Gly1961Glu)
CIC08381	4722	Index	IVS49	c.6816+1G>T	p.?	14	c.2023G>A [38]	p.(Val675Ile)			
CIC08383	4722	Affected sister	IVS49	c.6816+1G>T	p.?	14	c.2023G>A [38]	p.(Val675Ile)			
CIC08402	4722	Unaffected mother	WILD TYPE			14	c.2023G>A [38]	p.(Val675Ile)			

^+^: CIC03678 is carrier of a variant of uncertain significance (c.2868C>A), and hence was excluded from genotype-phenotype analysis. ^++^: CIC08933, unaffected father of CIC08932 does not carry any likely pathogenic variant therefore variant c.4775G>A could be de novo or CIC08933 is not the biological father of CIC08932. p.?: Protein has not been analysed, an effect is expected but difficult to predict.

**Table 3:** Clinical criteria used to classify patients in our study. FP: fundus photography; SW-AF: short-wavelength fundus autofluorescence; NIR-AF: near-infrared fundus autofluorescence; ERG: electroretinogram; OCT: optical coherence tomography; RPE: retinal pigment epithelium; EZ: ellipsoid zone; FS: foveal sparing.

	Groups/Stages	Criteria	Reference
Age of onset	n.a.	Age at which visual loss was first noticed	Lois et al., 2001 [53]
FP	Stage 1	Central macular atrophy with parafoveal or perifoveal flecks	Fishman GA et al., 1976 [54]
	Stage 2	Numerous flecks extended anterior to the vascular arcades and/or nasal to the optic disc	
	Stage 3	Desorbed flecks with choriocapillaris atrophy within the macula	
	Stage 4	Widespread RPE and chorioretinal atrophy throughout the fundus defined stage	
SW-AF and NIR-AF	Group 1	Central lesion with jagged border	Duncker et al., 2014 [55]
	Group 2	Lesion with extensive fundus changes	
	Group 3	Central lesion with smooth border and hyperautofluorescent SW-AF and NIR-AF ring	
	Group 4	Central lesion with smooth border and no hyperautofluorescent NIR-AF ring	
	Group 5	Discrete central lesions better visualized in NIR-AF images	
	Peripapillary area preserved	No alterations within an eccentricity of 0.6 mm from the optic disc	Cideciyan et al., 2005 [56]
	Flecks in the peripapillary area	Presence of flecks within an eccentricity of 0.6 mm from the optic disc	
	Peripapillary area not preserved	Absence of EZ band and/or EPR atrophy within an eccentricity of 0.6 mm from the optic disc	
ERG	I	Normal scotopic and full-field ERG	Lois et al., 2001 [53]
	II	Loss of photopic function	
	III	Loss of both photopic and scotopic function	
SW-AF and OCT	FS-YES	Foveal sparing	Fujinami et al., 2013 [38]
	FS-NO	Early onset foveal atrophy	
OCT	EZ Absent	EZ band loss	Parodi et al., 2015 [57]
	EZ Disrupted	EZ band disorganization	
	EZ Preserved	Identification of EZ band	
Genotype	NM	At least one null or splice variant is present	Fujinami et al., 2013 [52]
	MM	Two or more missense variants are present	

## Supplementary Materials

Supplementary materials can be found at http://www.mdpi.com/1422-0067/19/8/2196/s1.

## Abbreviations

MDPI	Multidisciplinary Digital Publishing Institute
STGD-1	Stargardt disease-1
*ABCA4*	ATP-binding cassette, subfamily A, member 4
BCVA	Best Corrected Visual Acuity
ff-ERG	Full field electroretinography
mf-ERG	Multi focal electroretinography
SW-AF	Short-wavelength autofluorescence
NIR-AF	Near-infrared Autofluorescence
SD-OCT	Spectral Domain Optical Coherence Tomography
ISCEV	International Society for Clinical of Electrophysiology of Vision
ACMG	American College of Medical Genetics and Genomics
AMP	Association of Molecular Pathology
HGMD	Human Gene Mutation Database
ExAC	Exome Aggregation Consortium
PolyPhen2	Polymorphism Phenotyping
UCSC	University of California Santa Cruz
SIFT	Sorting intolerant from tolerant
RPE	Retinal pigment epithelium
CRT	Central retinal thickness
TMV	Total macular volume
VUS	Variant of uncertain significance
MAF	Minor allele frequency
NGS	Next-generation sequencing
WES	Whole exome sequencing
WGS	Whole genome sequencing
EZ	Ellipsoid zone
FP	Fundus photograph
FS	Foveal sparing
MM	At least one missense variant in the genotype
NM	At least one null variant in the genotype
